# Auto-Luminescent Genetically-Encoded Ratiometric Indicator for Real-Time Ca^2+^ Imaging at the Single Cell Level

**DOI:** 10.1371/journal.pone.0009935

**Published:** 2010-04-01

**Authors:** Kenta Saito, Noriyuki Hatsugai, Kazuki Horikawa, Kentaro Kobayashi, Toru Matsu-ura, Katsuhiko Mikoshiba, Takeharu Nagai

**Affiliations:** 1 Research Institute for Electronic Science, Hokkaido University, Kita-20, Nishi-10 Kita-ku, Sapporo, Hokkaido, Japan; 2 Research Center for Cooperative Projects, Hokkaido University, Kita-15, Nishi-7 Kita-ku, Sapporo, Hokkaido, Japan; 3 Laboratory for Developmental Neurobiology, Brain Science Institute, RIKEN, 2-1 Hirosawa, Wako City, Saitama, Japan; 4 Precursory Research for Embryonic Science, Japan Science and Technology Agency, Sanbancho, Chiyoda-ku, Tokyo, Japan; University of California, Berkeley, United States of America

## Abstract

**Background:**

Efficient bioluminescence resonance energy transfer (BRET) from a bioluminescent protein to a fluorescent protein with high fluorescent quantum yield has been utilized to enhance luminescence intensity, allowing single-cell imaging in near real time without external light illumination.

**Methodology/Principal Findings:**

We applied BRET to develop an autoluminescent Ca^2+^ indicator, BRAC, which is composed of Ca^2+^-binding protein, calmodulin, and its target peptide, M13, sandwiched between a yellow fluorescent protein variant, Venus, and an enhanced *Renilla* luciferase, RLuc8. Adjusting the relative dipole orientation of the luminescent protein's chromophores improved the dynamic range of BRET signal change in BRAC up to 60%, which is the largest dynamic range among BRET-based indicators reported so far. Using BRAC, we demonstrated successful visualization of Ca^2+^ dynamics at the single-cell level with temporal resolution at 1 Hz. Moreover, BRAC signals were acquired by ratiometric imaging capable of canceling out Ca^2+^-independent signal drifts due to change in cell shape, focus shift, etc.

**Conclusions/Significance:**

The brightness and large dynamic range of BRAC should facilitate high-sensitive Ca^2+^ imaging not only in single live cells but also in small living subjects.

## Introduction

Bioluminescent proteins such as luciferase are a powerful tool for monitoring biological processes including gene expression in living organisms since bioluminescent signals can be acquired without an external light source; bioluminescence imaging is thus completely free from phototoxicity, photo-induced physiological reaction, and autofluorescence from the specimen, enabling signal detection from deep inside the tissue with high signal-to-noise ratio. These properties make bioluminescent proteins potentially superior to fluorescent proteins as a bioimaging tool. However, bioluminescence signals are too dim to be measured in real time, i.e., bioluminescence imaging generally requires longer exposure (more than several tens of seconds) than fluorescence imaging that takes less than 1 second. To overcome this drawback, an auto-illuminating fluorescent protein, eBAF-Y, has been developed [1]. eBAF-Y is based on the highly efficient bioluminescence resonance energy transfer (BRET) between enhanced *Renilla reniformis* luciferase (RLuc8) [2] and enhanced yellow fluorescent protein (EYFP). eBAF-Y emits a 3.5-fold brighter signal than RLuc8 alone, enabling observation of subcellular structure at the single-cell level in near real time at 0.1 Hz. This strategy has been applied to develop a similar auto-illuminating fluorescent protein, BRET3, composed of a mutant red fluorescent protein (mOrange) and RLuc8 [3]. BRET3 exhibits red-shifted light output peaking at 564 nm, making it easier to observe biological phenomena deep inside a small animal. Aequorin, another well-known bioluminescent protein, emits luminescence peaking at 466 nm in the presence of Ca^2+^ so it has been used as a Ca^2+^ indicator to monitor free Ca^2+^ concentration ([Ca^2+^]) in living specimens [4]. The highly efficient BRET has also been applied to aequorin by means of fusion with green fluorescent protein (GFP), thereby increasing the luminescence intensity by about 50 fold [5]. Although GFP-aequorin is bright enough to allow real-time imaging, it is not suitable for long-term imaging since aequorin must be regenerated after emission upon Ca^2+^ binding, which takes more than several tens of minutes [6], [7]. To circumvent this weak point, a Ca^2+^ indicator was developed based on split RLuc and Gluc [8], [9]. Signal change of these indicators is based on complementation of the N- and C-terminal halves of the split luciferase via Ca^2+^-induced interaction between CaM and M13. Although these indicators can detect Ca^2+^-dependent signal changes in living cells, they could not calibrate [Ca^2+^] because imaging of these indicators is based on single-emission measurement and thus the signal intensity change is highly dependent on the stoichiometric composition of the N- and C-terminal halves of the split luciferase in the cells. Also, these indicators cannot cancel out signal drift mainly caused by change in cell shape, focus drift, and uptake and consumption of coelenterazine-h due to auto or catalytic oxidization. To allow quantitative long-term real-time Ca^2+^ imaging, we designed a Ca^2+^ indicator based on BRET between RLuc8 and Venus, a brighter version of EYFP [10]. As the Ca^2+^-sensing domain, we used a Ca^2+^ binding protein, calmodulin (CaM), and its target peptide, M13, as in the case of the well-known FRET-based Ca^2+^ indicator, cameleon [11]. Binding and release of Ca^2+^ from CaM induces reversible conformational change of the CaM-M13 fusion domain between an extended and compact form, which, in turn, changes the distance between Venus and RLuc8 whereby their emission is reciprocally changed due to BRET. Thus, this indicator enables ratiometric observation that can cancel out artifactual signal drift. To obtain a large BRET change, we tried several circularly permuted Venus variants [12] to optimize the relative dipole orientation between the coelenterazine in RLuc8 and the choromophore of Venus. The best one, which we named BRAC, has a wild-type Venus and showed 60% signal change upon Ca^2+^ binding. Due to the brightness and large dynamic range, we successfully visualized Ca^2+^ oscillation induced by agonist stimulation of Hela cells with improved time resolution and high signal-to-noise ratio.

## Results

### Construction of BRET-based reversible Ca^2+^ indicators

**Figure 1:**
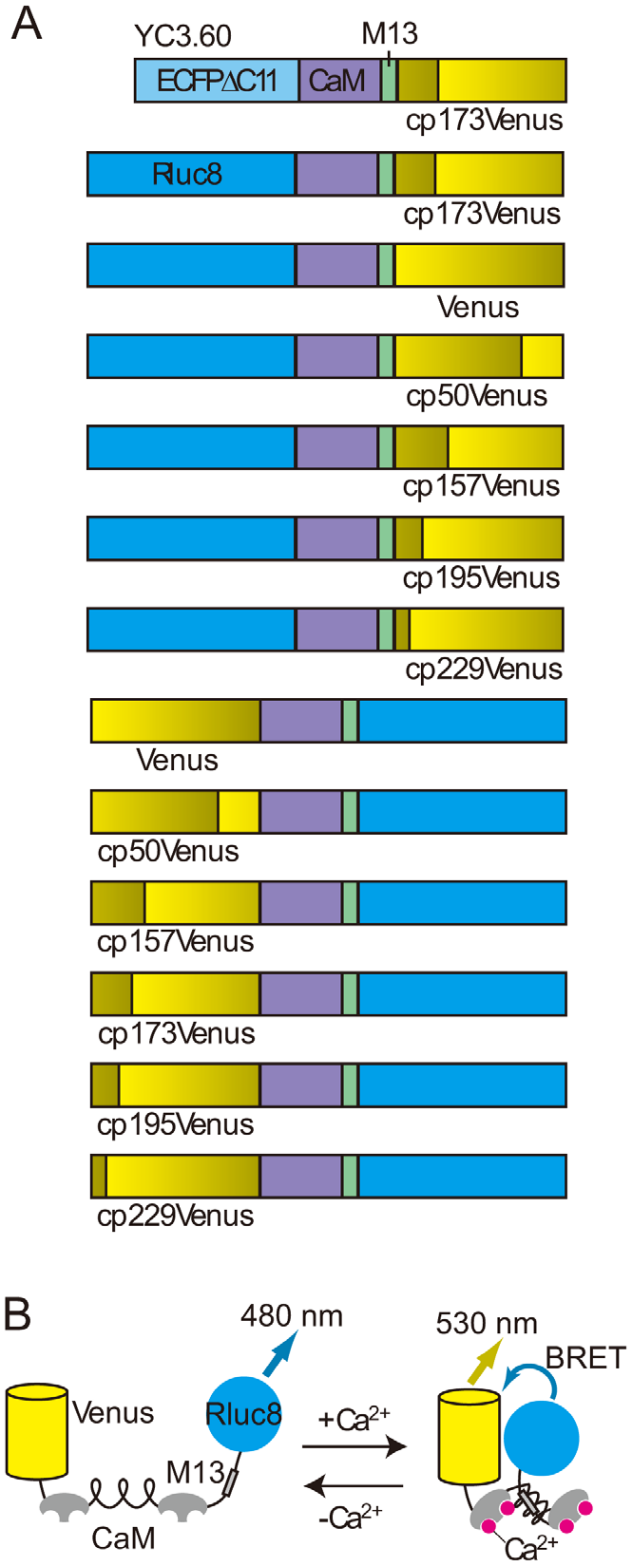
Schematic structures of yellow cameleon 3.60 (YC3.60) and BRAC derivatives. (**A**) Domain structure of YC3.60 and BRAC derivatives. Circularly permuted Venus variants are indicated as cp50, 157, 173, 195, and 229Venus, which have different translation start sites from the original Venus. (**B**) Schematic three-dimensional structure of BRAC, which is composed of the Ca^2+^-sensing domain, calmodulin (CaM) and M13, sandwiched between Venus and RLuc8. In the Ca^2+^ free state (left panel), CaM-M13 has an extended conformation so Venus is not located close to RLuc8 and thus only weak emission can be seen from Venus due to low BRET efficiency. Upon Ca^2+^ binding to CaM (right panel), Ca^2+^-CaM makes a compact complex with M13, which induces efficient BRET from RLuc8 to Venus resulting in fluorescence emission peaking at 530 nm.

**Figure 2:**
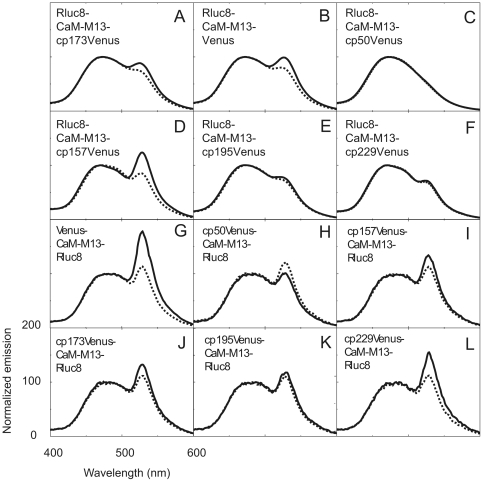
Spectral properties of BRAC derivatives. Emission spectra of BRAC derivatives, (**A–F**) RLuc8-CaM-M13-Venus or cpVenus variants, (**E–L**) Venus or cpVenus variants-CaM-M13-RLuc8, under zero Ca^2+^ (dashed line) and saturated Ca^2+^ (solid line) conditions.

To create a BRET-based autoluminescent reversible Ca^2+^ indicator, we chose the CaM-M13 chimeric protein for the Ca^2+^-sensing domain as in the case of the FRET-based Ca^2+^ indicator, cameleon YC3.60 (see Ref. 12). ECFP in YC3.60 was first replaced with RLuc8, the brightest version of luciferase [2], to yield RLuc8-CaM-M13-cp173Venus (Figure 1). The construct was bacterially expressed and purified for measuring the signal change in the peak intensity ratio (530/480 nm) with Ca^2+^. The dynamic range of the BRET signal change in RLuc8-CaM-M13-cp173Venus was 10% (Figure 2). Then, according to the strategy for expanding the dynamic range of FRET-based indicators [12], we replaced the Venus moiety in RLuc8-CaM-M13-Venus with a wild-type Venus or circularly permuted Venus variants that have a different translation start site from the original cp173Venus, as shown in Figure 1A. Among them, RLuc8-CaM-M13-Venus and RLuc8-CaM-M13-cp157Venus showed a 3-fold enhanced dynamic range (Figure 2). To further expand the dynamic range, we attempted to exchange the donor and acceptor in the above constructs to place the donor and acceptor molecule at the C- and N-terminus, respectively, of CaM-M13 (Figure 1A). Of these constructs, Venus-CaM-M13-RLuc8 had a 2-fold higher dynamic range than RLuc8-CaM-M13-Venus and RLuc8-CaM-M13-cp157Venus while other constructs showed a lower dynamic range (Figure 2). This result was consistent with previous results showing that orientation in which the donor luciferase was located at the C-terminus of the fusion generated a higher BRET signal compared to the reverse orientation [1], [13]. Overall, Venus-CaM-M13-RLuc8, which we designated as BRAC (BRet-based Autoluminescent Ca^2+^ indicator), had the largest dynamic range (60%) among the constructs that we tested (Table 1).

**Table 1:** Emission ratio (530 nm/480 nm) without and with Ca^2+^ and dynamic range of newly developed constructs.

Construct	− Ca^2+^	+ Ca^2+^	Dynamic range (%)
Rluc8-CaM-M13-cp173Venus	0.8	0.9	10
Rluc8-CaM-M13-Venus	0.8	1.0	30
Rluc8-CaM-M13-cp50Venus	0.5	0.5	0
Rluc8-CaM-M13-cp157Venus	0.9	1.2	30
Rluc8-CaM-M13-cp195Venus	0.7	0.8	10
Rluc8-CaM-M13-cp229Venus	0.7	0.7	0
Venus-CaM-M13-Rluc8	1.1	1.8	60
cp50Venus-CaM-M13-Rluc8	1.2	1.0	20
cp157Venus-CaM-M13-Rluc8	1.1	1.4	20
cp173Venus-CaM-M13-Rluc8	1.1	1.3	10
cp195Venus-CaM-M13-Rluc8	1.1	1.2	10
cp229Venus-CaM-M13-Rluc8	1.1	1.5	30

### Physicochemical properties of BRAC

**Figure 3:**
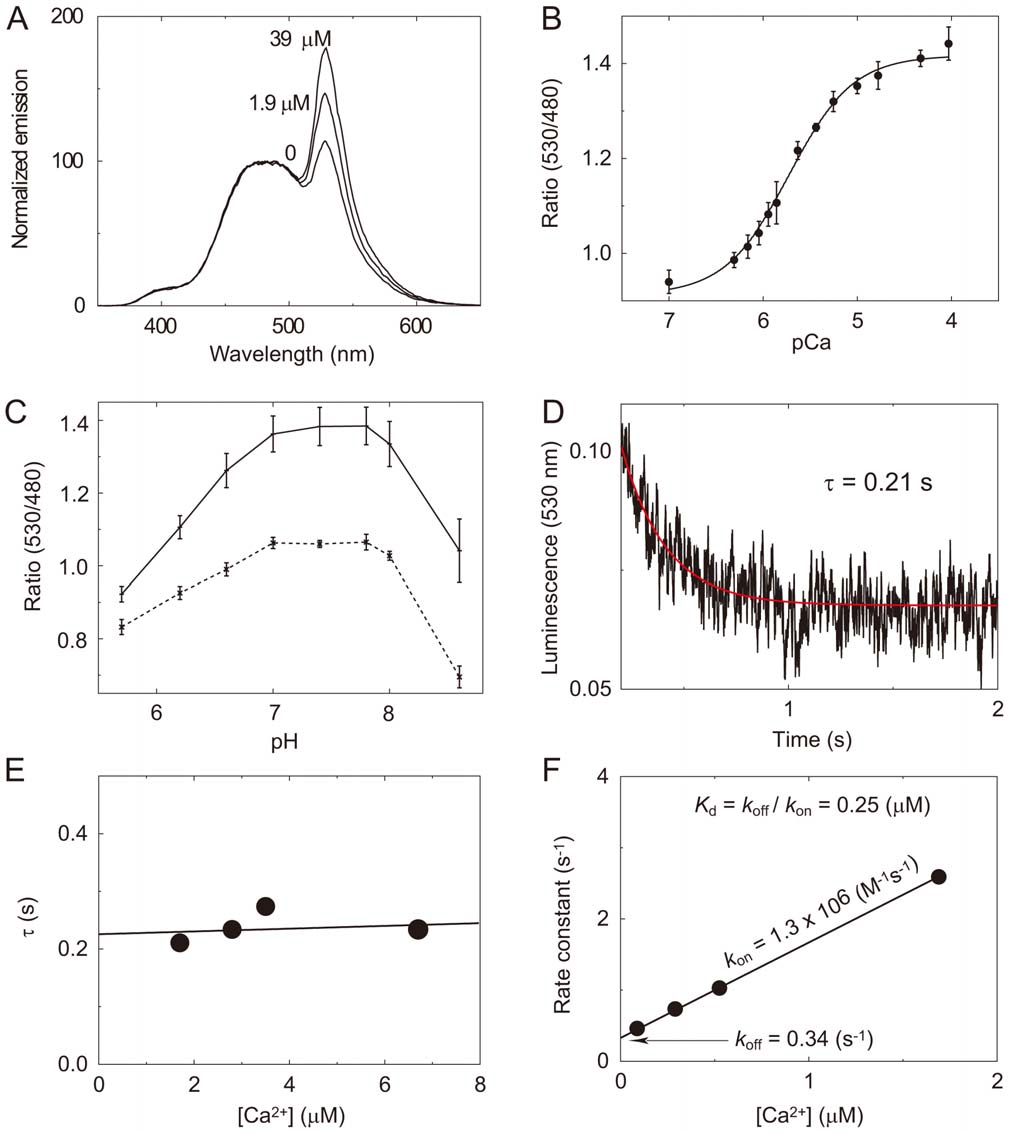
Chemical properties of BRAC. (**A**) Normalized emission spectra of BRAC at indicated free Ca^2+^ concentrations. All spectra were normalized at 480 nm. (**B**) Ca^2+^ titration curve of emission ratio (530 nm/480 nm) in BRAC. (**C**) pH titration curves of BRAC. pH titration curves of BRAC at zero (dashed line) and saturated (solid line) Ca^2+^. (**D**) Time traces of luminescence intensity of BRAC after the rapid mixing of Ca^2+^-saturated BRAC with Ca^2+^-free buffer. A fitted curve of single-exponential function is shown as red lines. (**E**) The dissociation time constant at various Ca^2+^ concentration. (**F**) Relaxation rate constant (*k*_obs_) for reaction of YC3.60 with Ca^2+^. Association rate (*k*_on_) and dissociation rate (*k*_off_) can be calculated from fitting equation *k*_obs_ = *k*_on_ [Ca^2+^] + *k*_off_ and dissociation constant (*K*_d_) can be calculated from the following equation *K*_d_ = *k*_off_/*k*_on_.

To evaluate the chemical properties of BRAC, Ca^2+^ titration was carried out. As shown in Figure 3A, emission from acceptor Venus increased in accordance with [Ca^2+^] while emission from donor RLuc8 was almost constant. This result closely corresponds to the result from the BRET-induced luminescence enhancement in BAF-Y as reported previously [1]. Ca^2+^ titration revealed that BRAC gave a monophasic Ca^2+^ response curve with a Hill coefficient of 1.3, and apparent dissociation constant of 1.9 µM (Figure 3B). We also perform pH titration of BRAC, which indicated that the emission ratio of 530 to 480 nm in BRAC was stable in physiological pH between 6.5 and 8.0 (Figure 3C). We then measured the Ca^2+^-association kinetics of BRAC by stopped-flow photometry system. However, the time course data we obtained was composed of at least two exponential decay components (*ι*<0.1 sec) which were thought to be derived from both Ca^2+^ and coelenterazine-h binding to BRAC. Because both kinetics were quite similar, we could not distinguish which component was derived from Ca^2+^ binding to BRAC. Thus, we then measured Ca^2+^-dissociation kinetics of BRAC (Figure 3D). The dissociation time constant of BRAC (*ι* = 0.21 sec) was independent on Ca^2+^ concentration and much smaller than that of YC3.60 (ι = 2.9 sec, *k*_off_ = 0.34 s^−1^) (Figure 3E, F) and comparable to that of G-CaMP (*ι* = 0.19 sec) [14]. This result was in good agreement with the fact that Ca^2+^ affinity of BRAC (*K*_d_ = 1.9 µM) is about 10 times lower than that of YC3.60 (*K*_d_ = 0.25 µM), and indicated that association/dissociation kinetics of BRAC are fast enough to detect Ca^2+^ dynamics in living cells and organelles.

### Ca^2+^ imaging by BRAC in living Hela cells

**Figure 4:**
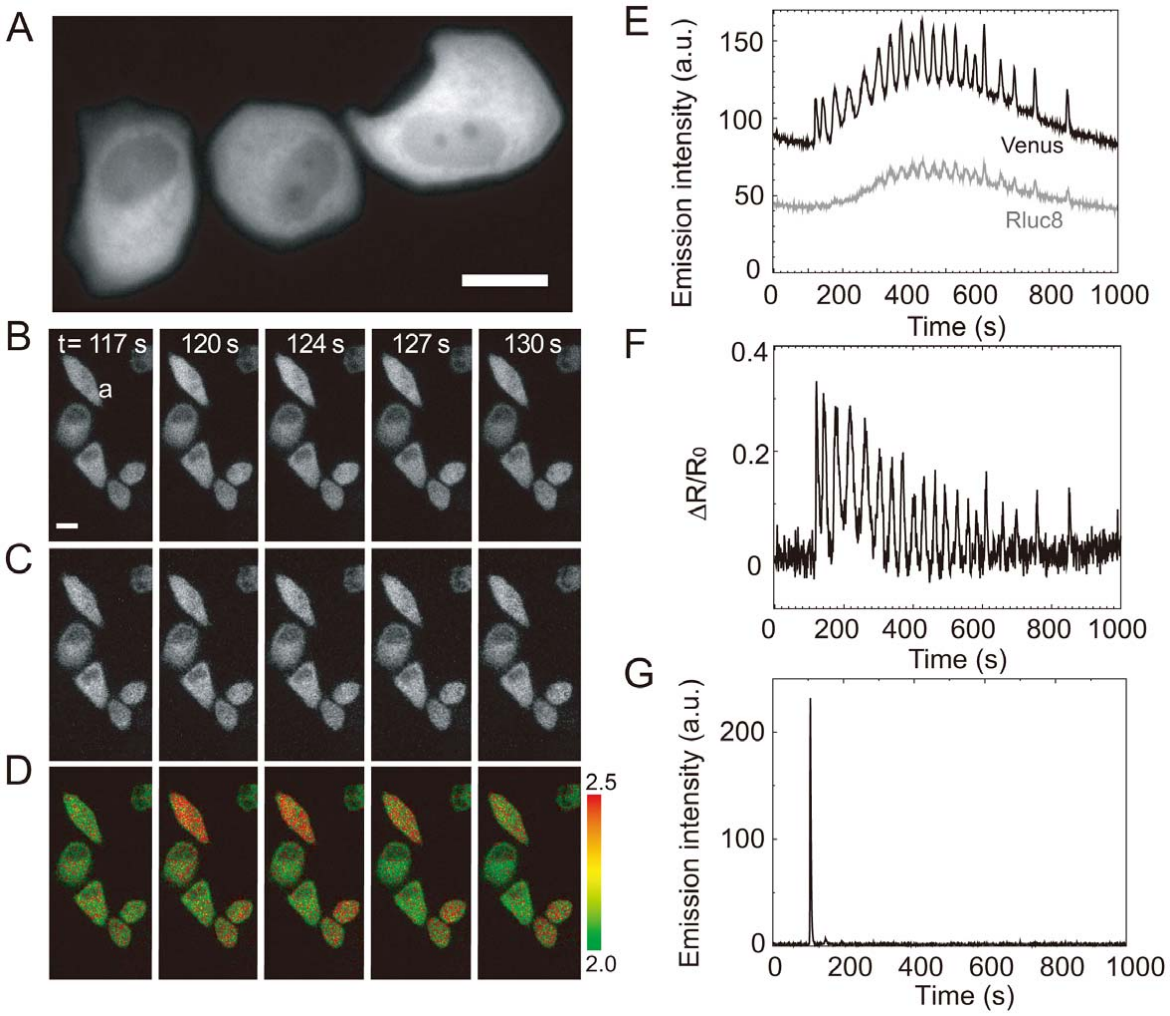
Ca^2+^ imaging of Hela cells by using BRAC. (**A**) Fluorescence image of Hela cells expressing BRAC. Emission signal from Venus (**B**) and RLuc8 (**C**). (**D**) A series of pseudo-colored BRET images showing Ca^2+^ dynamics taken every 1 second. (**E**) Time course of averaged intensity of RLuc8 (gray line) and Venus (black line) at a cell shown in (B). (**F**) Time course of ΔR/R_0_ at the same cell. (**G**) Time course of averaged intensity of Hela cell expressing G5A. 10 µM histamine treatment were done at ∼100 second. Scale bar, 20 µm.

In order to determine the validity of BRAC in living cells, we transfected Hela cells with the cDNA encoding BRAC. Direct excitation of the Venus moiety with blue light revealed that BRAC was localized in the cytoplasmic compartment, and slightly in the nucleus (Figure 4A). Figure 4B, 4C and 4D show the time course of RLuc8 and Venus, and pseudo-colored Venus/RLuc8 ratio images, respectively (see Video S1). Upon stimulation with 10 µM histamine, RLuc8 signals did not show a spike-like shape and sometimes increased or decreased slightly while Venus signals showed an oscillated spike after histamine stimulation as well as drifting of basal signal intensity, as seen in RLuc8 signals (Figure. 4E). The drift observed in both RLuc8 and Venus signals might be caused by the uptake and consumption of coelenterazine-h or the change in cell shape. However, due to the ratiometric imaging of BRAC, this signal drift was successfully canceled out by dividing the Venus signal with that of RLuc8 (Figure 4F). We also examined the dynamic range of BRAC in Hela cells by measuring minimum and maximum BRET ratio values which were obtained by treatment of HeLa cells with ionomycin/1 mM EGTA and ionomycin/5 mM CaCl_2_, respectively. The dynamic range obtained was 44% which was comparable to that measured *in vitro*.

For further validation of BRAC as an auto-luminescent Ca^2+^ indicator, we compared performance of BRAC with other BRET-based Ca^2+^ indicator, G5A [5] in HeLa cells. The signal to noise ratio of G5A was much better than that of BRAC (Figure 4G) because G5A did not emit any luminescence before Ca^2+^ binding. However, G5A failed to report histamine-induced Ca^2+^ oscillations that were reproducibly observed by using BRAC (Figure 4F, 4G), probably due to much slow regeneration rate of aequorin from apoaequorin [6], [7]. This result indicates the superiority of BRAC in terms of long term imaging of Ca^2+^ dynamics.

### Ca^2+^ imaging by BRAC in plant tissue

**Figure 5:**
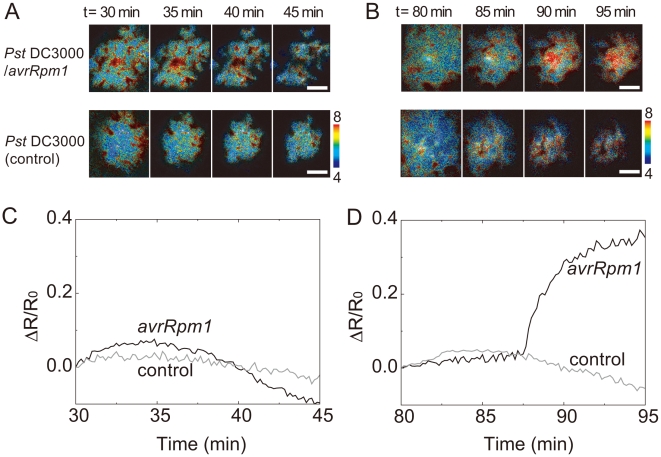
Ca^2+^ imaging of plant leaves by using BRAC. A series of pseudo-colored BRET images showing Ca^2+^ dynamics in plant leaves 30 minutes (**A**) and 80 minutes (**B**) after inoculation of *Pst* DC3000/*avrRpm1* and *Pst* DC3000 (control). (**C, D**) Time course of ΔR/R_0_ at the plant leaves inoculated *Pst* DC3000/*avrRpm1* (black line) and *Pst* DC3000 (gray line). 0 min represents the time of bacteria inoculation. Scale bars, 1 mm.

To further examine BRAC performance, we tried Ca^2+^ imaging in plant leaves, in which fluorescence-based Ca^2+^ imaging is difficult owing to the chloroplasts-derived strong autofluorescence, which disturbs reliable detection of fluorescence signal from Ca^2+^ indicator. Ca^2+^ is a ubiquitous intracellular second messenger involved in numerous signaling pathways in plants. Elevation of cytosolic Ca^2+^ concentration ([Ca^2+^]_i_) are common early event in plant defense signaling, and Ca^2+^ plays a essential role in activating the plant's surveillance system against attempted microbial invasion [15]. *Arabidopsis thaliana* has resistance to *Pseudomonas syringae* pv. *Tomato* (*Pst*) DC3000 having the avirulence gene *avrRpm1* (*Pst* DC3000/*avrRpm1*) but susceptible to *Pst* DC3000 [16]. To visualize the [Ca^2+^]_i_ increase during the plant defense signaling, we inoculated bacterial pathogens into the leaves of BRAC-expressing *Arabidopsis* lines. We monitored the changes in [Ca^2+^]_i_ during 30–45 and 80–95 minutes after the bacterial inoculation (Figure 5). Increase in [Ca^2+^]_i_ was observed at approximate 90 minutes after the inoculation of *Pst* DC3000/*avrRpm1* but not *Pst* DC3000. This result is consistent with the finding performed using aequorin-expressing *Arabidopsis* plants [17]. In that paper, aequorin could monitor [Ca^2+^]_i_ change in whole leaves every 5 sec or visualize [Ca^2+^]_i_ change in single leaf every 50 minutes. On the other hand, BRAC successfully visualized the [Ca^2+^]_i_ change in a region of single leaf every 10 sec.

## Discussion

We successfully designed and constructed the BRET-based ratiometric Ca^2+^ indicator, BRAC, which could visualize intracellular Ca^2+^ dynamics with 1-second temporal resolution at the single-cell level. Taking advantage of the bioluminescence imaging property that does not require external excitation light, BRAC is a potentially powerful tool for elucidating the cellular implementation of systems-level neural processes such as action, thought and emotion by combining its use with an optogenetic technology for genetically targeted, millisecond-timescale optical excitation of neurons expressing a light-driven cation channel and pump such as Channelrhodopsin-2, and chloride pump, NpHR, respectively [18].

To date, many FRET-based indicators have been developed [19]. Generally, these indicators use CFP and YFP as the FRET donor and acceptor, respectively. Given the similar emission spectrum between CFP and RLuc8, we could construct BRET-based indicators simply by substituting RLuc8 for the CFP moiety in the FRET-based indicators. However, this substitution of donor molecule may not yield high-performance indicators with a large dynamic range. To enhance the dynamic range, it may be necessary to use circularly permuted Venus variants to enable optimization of relative orientation between RLuc8 and Venus. Also, the development and application of circularly permuted RLuc8 variants might greatly improve BRET-based indicators in future.

## Materials and Methods

### Reagents

Coelenterazine-h was purchased from Promega (Madison, WI), Ni-NTA agarose from Qiagen GmbH (Hilden, Germany), Lipofectamine 2000 from Invitrogen (Carlsbad, CA), Dulbecco's Modified Eagle's Medium (DMEM) from Sigma (St. Louis, MO), ionomycin from Calbiochem (San Diego, CA) and fetal bovine serum from BioWest (Nuaillé, France).

### Gene construction

pGL4.70 [hRLuc] (Promega) was used as the template for introducing eight mutations, A55T/C124A/S130A/K136R/A143M/M185V/M253L/S287L, by means of a site-directed mutagenesis method to yield RLuc8 [2]. A series of BRET-based Ca^2+^ indicators was constructed by replacing ECFP and cp173Venus in YC3.60 with Venus or circularly permuted Venus variants, and RLuc8, respectively. G5A gene was constructed by fusion of GFP gene with aequorin coding sequences through 5 times repeating SGGSGSGGQ peptide coding sequence as shown previously [5].

### Protein expression, purification and Ca^2+^ titration in vitro

Recombinant BRAC protein with N-terminal polyhistidine tags was expressed in *Escherichia coli* [JM109(DE3)] at 23°C, purified using an Ni-NTA column (Qiagen). Emission spectra of BRAC were measured using a spectrophotometer (F-2500; Hitachi) and a microplate reader (SH-9000; Corona Electric). Final concentration of 1–10 µM coelenterazine-h (Promega) was used as the luminescent substrate for RLuc8. Ca^2+^ titration was performed by reciprocal dilution of Ca^2+^-free and Ca^2+^-saturated buffers prepared using *O,O*′-bis(2-aminoethyl)ethyleneglycol-*N,N,N′,N′-*tetraacetic acid (EGTA), *N*-(2-Hydroxyethyl)ethylenediamine-*N,N',N'*-triacetic acid (EDTA-OH), or Nitrilotriacetic acid (NTA) in 100 mM KCl, 10 mM MOPS (pH 7.2). Free Ca^2+^ concentrations were calculated by using 0.15, 4.3 and 170 µM as the *K*_d_ value of EGTA, EDTA-OH and NTA for Ca^2+^, respectively [20]. A Ca^2+^ titration curve was used to calculate apparent *K*_d_ value by non-linear regression analysis. The averaged data from eight independent measurements were fitted to the Hill equation using Origin7 software (OriginLab).

### Measurement of Ca^2+^ binding kinetics

Measurements of Ca^2+^ binding kinetics of BRAC were performed by using stopped-flow photometry system consisting of RX.2000 rapid mixing stopped-flow unit (Leatherhead, UK) and FP-750 spectrophotometer (JASCO, Japan). Emission intensity of Venus (530 nm) from BRAC were monitored at 1 kHz just after rapid mixing of 5 nM BRAC protein with 20 µM coelenterazine-h in various concentration of Ca^2+^ buffer. In this experiment, we did not mix coelenterazine-h with BRAC prior to measurement to avoid undesirable consumption of coelenterazine-h by Rluc8 in BRAC during sample preparation. Thus, time course of emission intensity in the stopped-flow experiments consists of three components of kinetics derived from Ca^2+^ binding to BRAC, coelenterazine-h binding to BRAC, and catalytic oxidation of coelenterazine-h by BRAC. To estimate the catalytic oxidation of coelenterazine-h by Rluc8 in BRAC, we measured time course of emission intensity change after mixing BRAC with 20 µM coelenterazine-h in Ca^2+^-free solution, and used the obtained data as a “base line”. Then, we measured time course of both association and dissociation of Ca^2+^ to and from BRAC by mixing 1 volume of BRAC in Ca^2+^-free buffer with 25 volume of solution containing 1.69 µM Ca^2+^, and 1 volume of BRAC in 1.69 µM Ca^2+^ solution with 25 volume of Ca^2+^-free buffer, respectively. The averaged data from at least 5 independent measurements were used for following analysis. The averaged time course data for association and dissociation kinetics were subtracted by the base line to remove the fraction derived from autonomous catalytic oxidation of coelenterazine-h by BRAC. Then, the time constants (ι) were calculated by means of curve fitting in single exponential equation using the data from 0.2 sec to 2.0 sec to minimize contribution of signal derived from association of coelenterazine-h with BRAC just after mixing. Measurements of Ca^2+^ binding kinetics of YC3.60 were performed as shown previously [11]. In the stopped-flow experiment, final Ca^2+^ concentration was controlled by reciprocal dilution of Ca^2+^-free and Ca^2+^-saturated buffers prepared using *O,O*′-bis(2-aminoethyl)ethyleneglycol-*N,N,N′,N′-*tetraacetic acid (EGTA) in 100 mM KCl, 10 mM MOPS (pH 7.2). Free Ca^2+^ concentration in every solution was confirmed with Ca^2+^-sensitive electrode (Metrohm, Herisau, Switzerland) which is calibrated with a CaCl_2_ standard solution (Orion Research, Cambridge, MA).

### pH titration

pH titration was performed using a series of 20 mM buffers with 100 mM KCl in pH 5.7 and 6.2 (MES), 6.6 and 7.0 (MOPS), 7.4 (Tris), 7.8 and 8.0 (HEPES), and 8.6 (Glycin).

### Cell culture and transfection

Hela cells were cultured in a homemade 35-mm glass-bottom dish in DMEM (Sigma) containing 10% fetal bovine serum (BioWest). Cells were transfected with plasmids by means of Lipofectamine 2000 (Invitrogen). At 1 to 2 days after transfection, cells expressing BRAC or G5A were subjected to imaging. 10 µM coelenterazine-h were added to the culture medium just before observation of BRAC and 1–4 hours before observation of G5A.

### Plant growth and pathology test conditions

We used homozygous *Arabidopsis thaliana* (ecotype Columbia, Col-0) expressing BRAC under control of the *35S* promoter. Plants were grown at 22°C under the 8-h-light in a day for 5–6 weeks. For pathology tests, we used *Pseudomonas syringae* pv. *tomato* DC3000 (*Pst* DC3000) and an avirulent strain (*Pst* DC3000/*avrRpm1*) that was kindly provided by Dr. Jeffery L. Dangl. We cultured the bacteria in KB medium with kanamycin (30 µg/ml) and rifampicin (100 µg/ml) [21]. After washing the cells twice in 10 mM MgCl_2_, we suspended them at 5×10^7^ cfu/ml for pathology tests. We inoculated the suspensions into the abaxial surfaces of leaves with needleless syringes.

### Live cell Imaging

An inverted microscope (Ti-E, Nikon) was used to observe BRAC and G5A signals in living cells maintained at 37°C with a continuous supply of 95% air and 5% carbon dioxide by using a stage-top incubator (Tokai Hit, Fujinomiya, Japan). Light emission from the samples was focused by a 40× oil-immersion objective lens (Nikon Plan Fluor, numerical aperture 1.3) on a cooled electron-multiplying charge-coupled device (EM-CCD) camera (ImagEM; Hamamatsu Photonics). A 2×2 binning setting was used for every 1-second imaging. To simultaneously acquire both RLuc8 and Venus images, we used a W-View system (Hamamatsu Photonics) composed of 510-nm short pass and 510-nm long pass dichroic mirrors, and F01-479/40 and F01-536/40 bandpass filters (both from Semrock) to split the RLuc8 and Venus images. Image analysis was performed using Aquacosmos (Hamamatsu Photonics). Maximum and minimum BRET ratio values in Hela cells were measured after treatment with 5 µM ionomycin/1 mM EGTA, and 5 µM ionomycin/5 mM Ca^2+^, respectively.

### Live plant imaging

We used macro zoom microscopy (MVX10, Olympus) with 1× objective lens (Olympus MVPLAPO1X, numerical aperture 0.25) on a cooled electron-multiplying charge-coupled device (EM-CCD) camera (iXon DU-897E-BV, Andor Technology). A 1×1 binning setting was used for every 10-second imaging. To simultaneously acquire both RLuc8 and Venus images, we used W-View system and mirrors as aforementioned in section of living cell imaging.

### Accession numbers

GenBank accession number for BRAC is GU180352.

## Supporting Information

Video S1 Video showing cytosolic Ca2+ oscillation in HeLa cells expressing BRAC in response to the application of 10 (micro)M hisitamine. Video corresponds to the data in Figure 4D.
(3.60 MB AVI)
